# Machine learning identifies novel markers predicting functional decline in older adults

**DOI:** 10.1093/braincomms/fcab140

**Published:** 2021-06-26

**Authors:** Kate E Valerio, Sarah Prieto, Alexander N Hasselbach, Jena N Moody, Scott M Hayes, Jasmeet P Hayes, for the Alzheimer’s Disease Neuroimaging Initiative

**Affiliations:** 1 Department of Psychology, The Ohio State University, Columbus, OH 43210, USA; 2 Chronic Brain Injury Initiative, The Ohio State University, Columbus, OH 43210, USA

**Keywords:** ADNI, angular gyrus, everyday cognition, IADL, machine learning

## Abstract

The ability to carry out instrumental activities of daily living, such as paying bills, remembering appointments and shopping alone decreases with age, yet there are remarkable individual differences in the rate of decline among older adults. Understanding variables associated with a decline in instrumental activities of daily living is critical to providing appropriate intervention to prolong independence. Prior research suggests that cognitive measures, neuroimaging and fluid-based biomarkers predict functional decline. However, *a priori* selection of variables can lead to the over-valuation of certain variables and exclusion of others that may be predictive. In this study, we used machine learning techniques to select a wide range of baseline variables that best predicted functional decline in two years in individuals from the Alzheimer’s Disease Neuroimaging Initiative dataset. The sample included 398 individuals characterized as cognitively normal or mild cognitive impairment. Support vector machine classification algorithms were used to identify the most predictive modality from five different data modality types (demographics, structural MRI, fluorodeoxyglucose-PET, neurocognitive and genetic/fluid-based biomarkers). In addition, variable selection identified individual variables across all modalities that best predicted functional decline in a testing sample. Of the five modalities examined, neurocognitive measures demonstrated the best accuracy in predicting functional decline (accuracy = 74.2%; area under the curve = 0.77), followed by fluorodeoxyglucose-PET (accuracy = 70.8%; area under the curve = 0.66). The individual variables with the greatest discriminatory ability for predicting functional decline included partner report of language in the Everyday Cognition questionnaire, the ADAS13, and activity of the left angular gyrus using fluorodeoxyglucose-PET. These three variables collectively explained 32% of the total variance in functional decline. Taken together, the machine learning model identified novel biomarkers that may be involved in the processing, retrieval, and conceptual integration of semantic information and which predict functional decline two years after assessment. These findings may be used to explore the clinical utility of the Everyday Cognition as a non-invasive, cost and time effective tool to predict future functional decline.

## Teaser

Valerio et. al. used machine learning to study predictors of functional decline. They found that neurocognitive measures showed the best predictive ability compared to demographic, MRI, FDG-PET, and fluid biomarker measures. Findings further identified semantic information processing as a novel construct that may be critical for predicting decline in function.

## Graphical Abstract

**Graphical Abstract:**
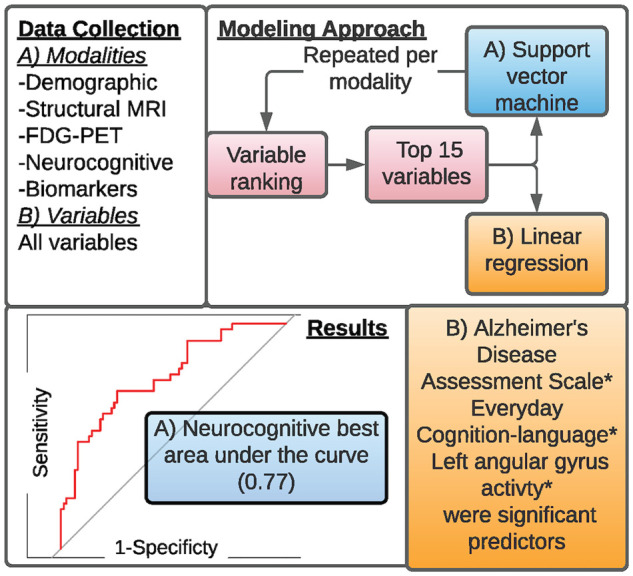

## Introduction 

The ability to carry out instrumental activities of daily living, such as paying bills, remembering appointments and shopping alone, decreases with age. An estimated 26% of community-dwelling older adults between 70 and 79 experience functional impairment in at least one domain (e.g. completing chores, managing money, preparing meals), increasing to 35% of adults between 80 and 89.^1^ Declines in IADLs are associated with Alzheimer’s disease,^2^ lower quality of life,^3^ depression^4^ and diminished physical functioning.^5^ However, IADL ability varies widely among older adults; whereas some show reduced functional independence, others demonstrate preserved functional independence. As the population continues to age and life expectancy increases,^6^ understanding variables associated with decline and preserved ability to carry out IADLs will be critical to provide appropriate and timely intervention.

A small set of studies have examined neurobiological and cognitive factors associated with longitudinal functional decline across the spectrum of normal and pathological ageing. One study demonstrated that CSF markers of neurodegenerative pathology – i.e. tau, phosphorylated-tau (p-tau) and amyloid beta (Aβ)-predicted functional decline at 36 months in cognitively normal subjects and individuals with mild cognitive impairment (MCI),^7^ while another study found that whole-brain atrophy based on structural MRI and Apolipoprotein E (*APOE)* ε4 status predicted functional decline in MCI.^8^ Fluorodeoxyglucose-PET and cognitive performance have also been shown to be associated with functional decline.^9–12^ Taken together, the emerging literature has outlined a variety of neurobiological and cognitive factors that are associated with subsequent functional decline. However, some important limitations characterize previous work. First, variables included in prediction analyses were selected *a priori* in these studies, potentially leading to overvaluation of some variables and oversight of others that may explain greater variance in functional decline. Second, prior work has not included testing/validation samples or employed permutation testing, limiting generalizability of findings. Finally, studies examining genetic associations of functional decline have conducted candidate gene analyses, rather than considering polygenic effects, which have been shown to add predictive value to understanding many complex traits and diseases.^13^

To address these limitations, we implemented machine learning techniques to identify variables at a baseline visit that were associated with a functional decline within 24 months later. This data-driven approach is designed to give equal consideration to all available variables and allows the relationships between variables to determine the final model. Such approaches prevent biased experimenter expectancies from influencing results and are ideal for studying a facet as highly dimensional as human ageing. Moreover, this technique can help identify risk and resilience factors from a large set of variables to allow a more targeted approach to treatment or prevention, providing a more individualized approach to understanding functional decline.^14^ Although prior research has used machine learning to diagnose and predict conversion to Alzheimer’s disease as well as Alzheimer’s disease pathology,^15–19^ this approach has yet to be implemented to examine functional decline. Functional decline is considerably more prevalent among older adults (∼26% of individuals between 70 and 79)^1^ compared to Alzheimer’s disease (9.7% of individuals over the age of 70),^20^ highlighting the need to examine variables predictive of functional decline.

In the current analyses, we implemented support vector machine (SVM) algorithms to construct models of functional decline at 24 months following baseline assessment using multiple data modalities (demographics, MRI, FDG-PET, neurocognitive and genetic/fluid-based biomarkers) available in the Alzheimer’s Disease Neuroimaging Initiative (ADNI) database. ADNI is a multicenter study that includes a sample of deeply characterized older adults. By grouping variables into modalities based on collection method, we sought to examine the extent to which less invasive methods of data collection (e.g. neurocognitive tests) have discriminative power equal to or better than more invasive markers of functional decline (e.g. CSF markers). In addition, we evaluated variables across modalities to identify the individual variables that were most associated with functional decline. Included in these variables was a polygenic risk score for Alzheimer’s disease, which takes into account the effects of many genes. Finally, we took the selected variables and validated their effect in a separate sample to provide evidence of the generalizability of their predictive ability. The goals of this study were 2-fold: (i) Use machine learning techniques to identify data modalities and variables most predictive of functional decline in an unbiased and data-driven way; and (ii) Test those variables in an independent sample and examine their influence on future decline using multiple regression models.

## Materials and methods

### Subjects

Data used for this manuscript were obtained from the ADNI database (adni.loni.usc.edu). ADNI is a large-scale longitudinal study that began in 2004 as a public–private partnership led by Principal Investigator Michael W. Weiner, MD. Participants were recruited to examine serial MRI, PET, biomarkers, clinical assessment and neuropsychological assessment to measure the progression of MCI and Alzheimer’s disease. Additional information can be found at adni.loni.usc.edu. Our analyses included 398 adults with preserved functional performance, as indicated by a diagnosis of normal cognition or MCI, and who completed the Functional Activity Questionnaire (FAQ) at a baseline and 24-month visit. Participants included in analyses were missing no more than 5% of all analysed variables. Remaining missing data were imputed via simple random sampling (<1% of total data).

Study procedures were approved by site-specific Institutional Review Boards and all participants and/or authorized representatives provided written informed consent consistent with the Declaration of Helsinki. For more information about the diagnostic criteria used in ADNI, see the General Procedures Manual at adni.loni.usc.edu.

### Data types

#### Outcome variable

The FAQ is a questionnaire that was designed to monitor functional changes in older adults and is considered the gold standard for assessing IADLs in an ageing population.^21^ It assesses ten items on a scale from 0 to 3, with higher scores indicating greater levels of impairment. Total scores range from 0 to 30. It has demonstrated high reliability and sensitivity (85%) in research and clinical settings^22^ and is useful for differentiating between cognitively normal and dementia patients,^22^ MCI and Alzheimer’s disease,^23^ and MCI and normal cognition.^3^ In order to capture subtle changes in functioning, individuals who had a difference score greater than or equal to one point between their baseline and 24-month assessment were considered to have a decrease in functioning. This corresponds with any decrease in ability to carry out any of the ten items assessed, and previous research has shown that any dependence on others for any IADL is significantly associated with increased risk of developing Alzheimer’s disease as early as five years prior to diagnosis.^24^ All other individuals were characterized as having either stable or improving functioning.

#### Predictor variables

A total of 508 variables were included in analyses, organized into five data modalities (demographics, MRI, FDG-PET, neurocognitive and genetic/fluid markers). These variables were also assessed for their individual contributions to functional decline. In total, there were 26 demographic variables, 341 MRI variables, 30 FDG-PET variables, 71 neurocognitive variables and 40 genetic/fluid-based biomarker variables.

#### Demographics

Twenty-six demographic variables included age, sex, education, race, ethnicity, marital status, body mass index, baseline diagnosis (CN or MCI) and medical history. Medical history diagnoses were classified into one of 18 categories of illnesses – psychiatric, neurologic, ears/nose/throat (ENT), cardiac, respiratory, hepatic, dermatologic, musculoskeletal, endocrine/metabolic, gastrointestinal, blood, renal, allergic, alcohol-related, drug-related, smoking related, malignant or surgical.

#### MRI

T_1_-weighted images were acquired on ADNI-approved 3 T scanners and processed cross-sectionally using the 2010 Desikan-Killiany atlas with FreeSurfer image analysis suite, version 5.1. For more information, see Supplementary material. This included 69 cortical volume measurements (68 unilateral volumes from the left and right hemispheres and intracranial volume), 16 hippocampal subfield volume measurements, 50 subcortical volume measurements, 70 surface area measurements and 136 thickness measurements. All volume measurements were corrected for intracranial volume.^25^

#### Fluorodeoxyglucose-PET

For more information on data collection and processing, see Supplementary material. Metrics of regions of interest (ROIs) available through ADNI were previously determined from a meta-analysis that identified regions which significantly differed between MCI and Alzheimer’s disease and predicted cognitive decline.^11^ Metrics of standardized uptake value in five regions were included: the right temporal gyrus, left temporal gyrus, right angular gyrus, left angular gyrus and posterior cingulate. Metrics of each region included the mean, median, mode, maximum, minimum and standard deviation SUV of each ROI, for a total of 30 variables. For these variables, SUV refers to the ratio of the concentration of radioactive glucose analog in an ROI to the concentration in the rest of the body, corrected for the weight of the patient. It is a proxy for glucose metabolism in the brain, with larger SUVs suggesting increased glucose uptake and increased brain activity.

#### Neurocognitive measures

General cognition, executive function, learning and memory, language, and processing speed were assessed with 13 standardized neuropsychological tests. Seventy-one neurocognitive variables were derived from these 13 tests; this included subtests when available in the NEUROBAT.csv file from ADNI. Supplementary Table 1 lists the specific tests used for each domain.

#### Biological samples

A single polygenic hazard score for Alzheimer’s disease was calculated using 31 single nucleotide polymorphisms and two *APOE* variants.^26^ CSF was collected via lumbar puncture and analysed for three variables: Aβ_1-42_, tau and p-tau. Blood plasma and urine serum were collected from participants and underwent standard clinical laboratory tests at the University of Pennsylvania Biomarker Core.^27^ Thirty-six variables from tests on blood plasma and urine serum were included in these analyses. For more information, see Supplementary material.

### Analytic methods

#### Training and testing sample

Analyses were run in R (3.6.1)^28^ using the ‘caret’ package.^29^ The sample was partitioned into two groups, a training sample (70% of the sample) and a testing sample (30% of the sample), using the createDataPartition function. This function assigned participants to one of two groups, while ensuring that both groups had equal proportions of individuals who were characterized as declining functionally. Training and testing samples were compared using Mann–Whitney U-tests for continuous variables and Fisher’s exact test for categorical variables. Nonparametric tests were used given that the properties required of parametric tests were not satisfied.

**Figure 1:**
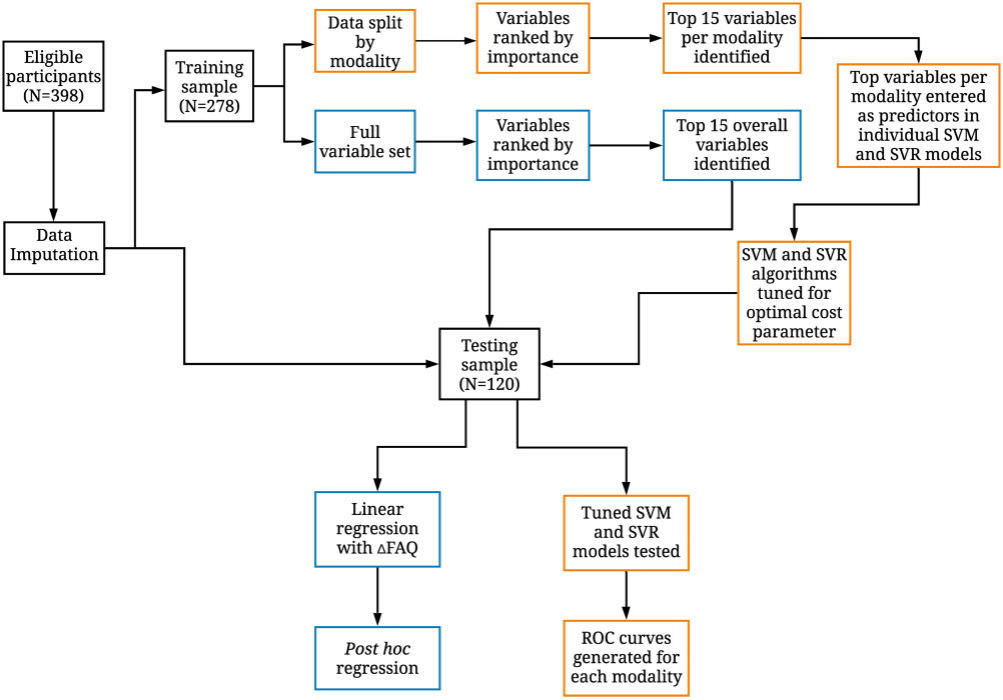
**Flowchart illustrating process of variable selection and data analysis.** Three hundred ninety-eight eligible participants were identified in the first step and missing data were imputed. In the second step, the data were split into the training sample and testing sample. Two parallel processes were run in the training sample. In the first process, shown in orange, data were split into each of the five modalities. Within each modality, the top 15 most predictive variables were identified by their individual variable importance. These selected variables were then entered as predictors into individual SVM models, one for each modality. Within these individual SVM models, the models were trained for the optimal cost parameter. This final model was then tested using the testing sample. In the testing sample, ROC curves were generated for each modality. In the second process in the training sample, shown in blue, all variables from all modalities were included together. The top 15 most predictive variables were identified by the individual variable importance. These selected variables were then entered as predictors into a linear regression in the testing sample. Also in the testing sample, *post hoc* hierarchical regression analyses were conducted to better understand the relationship between significant predictors. FAQ, Functional Activities Questionnaire; ROC, receiver operating characteristics; SVM, support vector machine.

Figure 1 shows the sequence of variable selection and variable evaluation. Briefly, the training sample was used to identify variables with the most predictive ability or highest variable importance as well as model tuning. The testing sample was used to evaluate model performance and generate receiver operating characteristic (ROC) curves, as well as the variance in functional decline explained by the selected predictor variables.

### Data modality analysis

#### Variable selection

To determine which of the five data modalities (i.e. demographics, MRI, FDG-PET, neurocognitive and genetic/fluid markers) best predicted functional decline, the top 15 variables from each modality were first identified by ranking their importance to the model’s prediction. Variable importance scores were calculated using the varImp function in the ‘caret’ package. For an SVM algorithm, this function works by considering individually each variable in a model and defining a line that most accurately separates the two classes, using SVM. See below for more information about how SVM defines a line or hyperplane. This classification boundary is then systematically varied to adjust the ratio of true positives to false positives. The resulting ratios are plotted to generate an ROC curve representing the trade-off between sensitivity and specificity for the variable. The area under the curve (AUC) thus summarizes the variable’s susceptibility to noise, with higher values suggesting greater discrimination ability. AUC was therefore used to determine variables with the greatest predictive ability. For more information about ROC curves and classification boundaries.^30^

#### Support vector machine

SVM classification algorithms were used to find the optimal solution to separate data into two different classes or categories. Briefly, SVMs function by plotting each input point in high-dimensional space and defining the optimal hyperplane that best separates the data into classes. The hyperplane is defined by finding certain data points at or near class boundaries (support vectors) and maximizing the distance (margin) between each support vector and the line separating them (the hyperplane). These analyses employed a linear kernel or a linear hyperplane to predict functional decline as a binary outcome 24 months after a baseline assessment. Variables identified during the variable selection step were used as predictors.

SVM classifiers were tuned for the optimum cost parameter prior to model evaluation in the testing sample. The cost parameter, *c*, is a regularization parameter, or the penalty for misclassification. The magnitude of *c* affects the size of the margin-hyperplane and can be tuned for the optimal trade-off between training errors and testing errors to optimize the model for generalizability. For these analyses, we tested several values of *c* (2^−8^, 2^−4^, 2^−2^, 1, 2, 16) to define the best model. The tuning process underwent cross-validation via repeated *k*-fold cross-validation.

For these analyses, *k* was set to 10 and 50 repeats were performed. This method of cross-validation works by randomly partitioning the sample into 10 (*k*) equal-sized groups. The first nine groups are used to train a model with a given value of a tuning parameter. The tenth group is then used to evaluate this model. A new model is then trained on nine of the groups while a different group is used for testing. This process continues until each group is used for model evaluation exactly once. The entire process is repeated 50 times for each value of the tuning parameter. This information was then used to select the model tuning parameters which were used for the final model that was evaluated in the testing sample.

Additionally, models were also run using the Synthetic Minority Oversampling Technique (SMOTE)^31^ to account for imbalance in the dataset. For more information, see Supplementary material and Supplementary Table 2.

#### Model evaluation

ROC curves were generated in the testing sample after variable selection and tuning in the training sample. Predictive ability of the models was assessed using AUC rather than accuracy because AUC has been shown to be a more consistent measure of evaluating models^32^ ; an AUC of 0.5 indicates no discrimination, 0.5 to 0.6 indicates poor discrimination, 0.7 to 0.8 indicates acceptable discrimination, 0.8 to 0.9 indicates excellent discrimination, and greater than 0.9 indicates outstanding discrimination. 

### Individual variable analysis

#### Variable selection

To determine which individual variables best predicted functional decline, the 15 variables (out of the 508 extracted from ADNI) with the highest variable importance scores were selected and retained for additional statistical analyses. This univariate variable selection method allowed us to significantly narrow the number of variables, from several hundred to 15. Doing so was conceptually suited for achieving the aims of this paper and permitted the interpretation of those variables through a clinical lens. As with data modality variable selection, variable importance scores were calculated by defining a line that most accurately separated the two classes; ROC curves were generated and AUCs were calculated. Doing so allowed the identification of the most predictive variables independent of data modality.

#### Regression analysis

Regression analysis was conducted using ΔFAQ as a semi-continuous outcome variable and the 15 variables with the highest individual AUC included as predictors. ΔFAQ was calculated as the difference between the baseline FAQ score and 24-month FAQ score. Higher scores indicated worse functioning at the follow-up visit relative to the baseline visit. To circumvent issues of collinearity and minimize variance inflation factor values, two variables were removed prior to regression analyses due to high correlations with other variables (maximum SUV of left angular gyrus, and EcogSP-Total). Because the mean SUV in the left angular gyrus encompasses the maximum SUV as well as additional information about activity in this region, the maximum SUV in the left angular gyrus was removed. The EcogSP-Total score is calculated as the average of all subtest scores. In order to include the most information, the EcogSP-Total was removed. The left subiculum volume and left CA2/3 volume were also highly correlated. Rather than choose one to remove, these two values were added together for the regression analysis. Supplementary Figure 1 reports the correlations between each of the 15 variables. For all statistical analyses, significance threshold was set at *P *<* *0.05.

### Data availability

Data used for these analyses are available by request at adni.loni.usc.edu.

## Results

### Demographic characteristics

Table 1 reports demographic characteristics of the participant sample. In total, 398 participants (53.8% male) between the ages of 55 and 90 (mean age = 71.4; SD = 6.9) were included in these analyses. These participants were randomly partitioned into a training sample (70%; *N *=* *278) and a testing sample (30%; *N *=* *120). The two groups did not differ significantly on age (*P *=* *0.77), sex (*P *=* *0.91), education (*P *=* *0.74), baseline FAQ score (*P *=* *0.30), ΔFAQ score (*P *=* *0.54) or proportion of participants with declining functioning (*P *=* *1.00).

**Table 1:** Demographic information and comparisons between the testing and testing sample

Variable	**All** **Mean (SD)**	**Training** **Mean (SD)**	**Testing** **Mean (SD)**	*P*-value
*N*	398	278	120	
Age	71.4 (6.9)	71.3 (6.7)	71.5 (7.4)	0.77
Males, *N* (%)	214 (53.8)	150 (54.0)	64 (53.3)	0.91
Education	16.5 (2.5)	16.5 (2.5)	16.4 (2.5)	0.74
Diagnosis, *N* (%)				0.94
Cognitively normal	128 (32.1)	91 (32.7)	37 (30.8)	
MCI	270 (67.8)	187 (67.3)	83 (69.2)	
Baseline FAQ^a^	1.9 (3.4)	1.8 (3.3)	2.1 (3.6)	0.30
Declining functioning, *N* (%)	133 (33.4)	93 (33.5)	40 (33.3)	1.00
ΔFAQ	1.4 (4.1)	1.6 (4.4)	1.1 (3.8)	0.54

a Score between 0 and 30, higher score indicates declining functioning.

FAQ, Functional Activities Questionnaire; MCI, mild cognitive impairment; SD, standard deviation.

### Most predictive data modality

**Figure 2:**
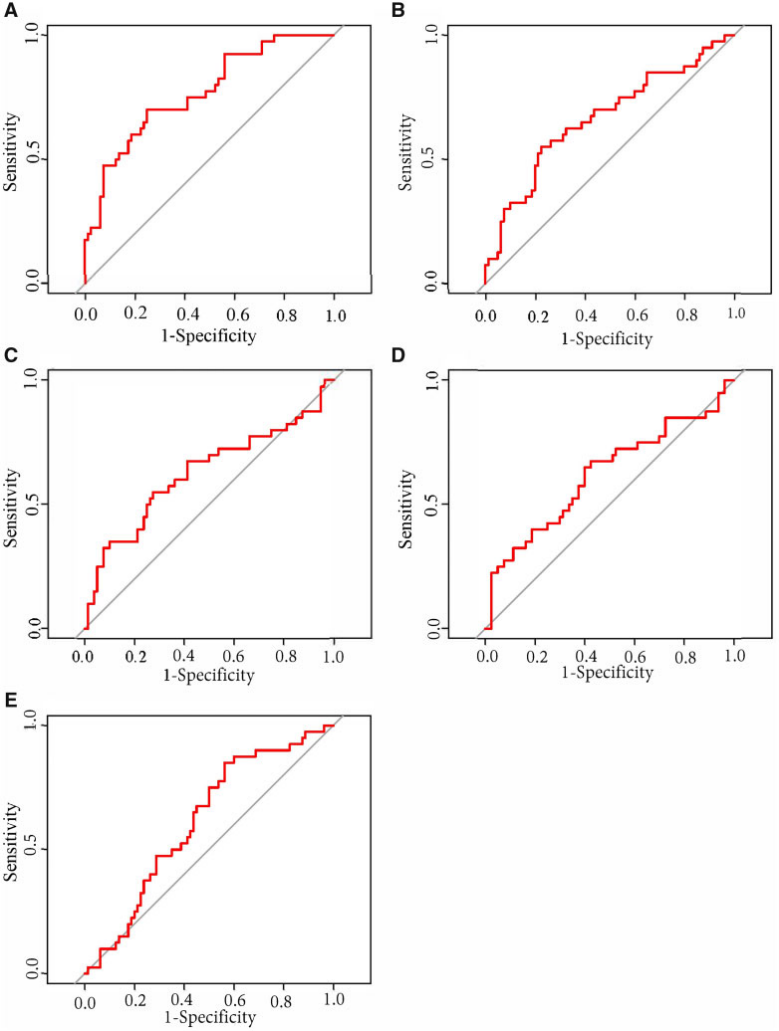
Classifier results. (**A**) Neurocognitive measures. (**B**) FDG-PET measures. (**C**) Genetics/fluid-based biomarkers. (**D)** MRI measures. (**E)** Demographic information. FDG, fluorodeoxyglucose.

Table 2 reports the variables with greatest AUC that were selected from each modality; Table 3 and Fig. 2 report model performance and confidence intervals of these variables in the testing sample. AUC ranged from poor (0.62) to acceptable (0.77). The best single-modality accuracy was achieved with neurocognitive measurements (Fig. 2A). This model demonstrated acceptable AUC (0.77, 95% CI = 0.68–0.86) and accuracy (74.2%). MRI metrics, FDG-PET metrics, genetic/fluid-based biomarkers and demographic information all demonstrated poor AUC, 0.62, 0.66, 0.63 and 0.62, respectively (Fig. 2D, B, C and E).

**Table 2:** Top ranked variables of each data modality as measured via individual AUC

Rank	Demographics^a^	MRI^a^	FDG-PET^a^	Neurocognitive^b^	Biomarkers^a^
1	Diagnosis (MCI or CN)	R hippocampus volume	Maximum L angular gyrus	CDR-sum of boxes	Amyloid beta
2	Psychiatric diagnosis	L CA2/3 volume	Mean L angular gyrus	EcogSP-Total	Albumin
3	Dermatological diagnosis	L subiculum volume	Median L angular gyrus	Logical memory delayed	Tau
4	Gastrointestinal diagnosis	L CA4/DG volume	Maximum BL cingulum post	EcogSP-Memory	p-tau
5	Malignancy	R CA2/3 volume	Max R angular gyrus	EcogSP-Divided Attention	PHS
6	Musculoskeletal diagnosis	R CA4/DG volume	Mean R angular gyrus	ADAS13	Total protein
7	Cardiac diagnosis	L hippocampus volume	Median BL cingulum post	MoCA	Percent eosinophils
8	Age	R subiculum volume	Mode R angular gyrus	EcogSP-Language	Neutrophils
9	Prior surgery	L entorhinal cortical thickness	Median R angular gyrus	RAVLT 30-min delay	Percent neutrophils
10	Education	L entorhinal cortical volume	Minimum R angular gyrus	Category fluency-animals	Serum glucose
11	Endocrine/metabolic diagnosis	R CA1 volume	Maximum L temporal gyrus	RAVLT % forgetting	Eosinophils
12	Smoking status	R entorhinal cortical thickness	Mean BL cingulum post	EcogSP-Visuospatial	Percent lymphocytes
13	Hematopoietic-Lymphatic diagnosis	R hippocampal tail volume	Median L temporal gyrus	RAVLT Trial 3	Basophils
14	Neurological diagnosis (Not AD)	L CA1 volume	Mean L temporal gyrus	RAVLT immediate	Gamma-glutamyl Transferase
15	Gender	L Presubiculum volume	Mean R temporal gyrus	RAVLT Trial 5	Percent monocytes

a Variables showed poor predictive value, see Table 3 for more information.

b Variables showed acceptable predictive value, see Table 3 for more information.

AD, Alzheimer’s disease; ADAS, Alzheimer’s Disease Assessment Scale; BMI, body mass index; CDR, Clinical Dementia Rating; CN, Cognitively normal; DG, Dentate gyrus; EcogSP, Everyday Cognition-Study Partner; ENT, Ears/Nose/Throat; FDG, fluorodeoxyglucose; L, left; MCI, mild cognitive impairment; MoCA, Montreal Cognitive Assessment; PHS, polygenic hazard score; p-tau, phosphorylated tau; R, right; RAVLT, Rey Auditory Verbal Learning Test.

**Table 3:** Model performance

Model	**Sensitivity**	**Specificity**	Accuracy (%)	AUC	**AUC** **95% CI**	C
Neurocognitive	0.48	0.88	74.2	0.77	0.68–0.86	2^–8^
FDG-PET	0.33	0.90	70.8	0.66	0.56–0.77	2^–2^
Genetic/fluid-based biomarkers	0.23	0.95	70.8	0.63	0.51–0.74	2^–4^
MRI	0.25	0.95	71.7	0.62	0.51–0.73	2^0^
Demographics	0.00	1.00	66.7	0.62	0.51–0.72	2^–8^
AUC, area under the curve; FDG, fluorodeoxyglucose.						

### Most predictive variable

The 15 variables with the highest individual AUCs from all 508 variables, listed alphabetically, were as follows: Alzheimer’s Disease Assessment Scale (ADAS13), Category fluency-animals, Clinical Dementia Rating-sum of boxes, Everyday Cognition-Study Partner (EcogSP)-Divided Attention, EcogSP-Language, EcogSP-Memory, EcogSP-Total, EcogSP-Visuospatial, left CA2/CA3 volume, left subiculum volume, Logical Delayed Memory, mean SUV of left angular gyrus, maximum SUV of left angular gyrus, Rey Auditory Verbal Learning Test 30-minute delay, right hippocampal volume and the total Montreal Cognitive Assessment score. These variables (with the exception of maximum SUV of the left angular gyrus and EcogSP-Total; see above) were then entered into a multiple regression model in the testing sample to predict ΔFAQ as a semi-continuous variable. Basic demographic variables were not added as covariates because they were not identified as significant predictors during variable selection.

Because the models demonstrated heteroskedasticity, a weighting parameter was used such that the absolute value of the residuals was regressed against each predictor. This improved heteroskedasticity. Furthermore, all models described met assumptions of a linear regression: the mean of the residual values was approximately zero; there was no autocorrelation of residuals; predictors and residuals showed no significant correlations; the predictors all showed positive variance; there was no multicollinearity present; and the residual values were approximately normally distributed. Additionally, because weighting parameters tend to lead to inflated *R*^2^ values, all reported *R*^2^ values have been adjusted as per Willet and Singer.^33^ This adjustment calculates *R*^2^ based on the difference between the model estimates and the original values and has been shown to be a less biased goodness of fit. Lastly, the final model included 12 predictors for 120 participants, which is in line with the recommendation of 1:10 predictor to participant ration for regression analysis.^34^^,^^35^

**Figure 3:**
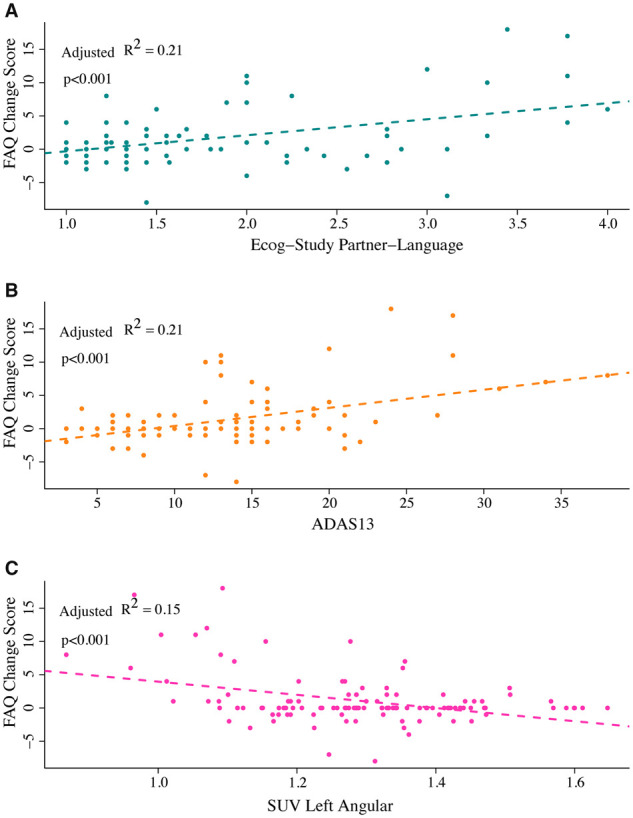
Relationship between significant predictors and ΔFAQ. (**A**) Values on the *x*-axis represent study partner report for language ability on the Everyday Cognition assessment, with higher scores indicating worse language ability. Scores are calculated as the average of the 9 items on the subtest. Values on the y-axis represent ΔFAQ, calculated as the difference between baseline FAQ score and 24-month FAQ score, with higher scores indicating worse functioning as the 24-month visit. Worse partner-reported language ability was associated with greater decline in functioning 24 months after baseline assessment. (**B**) Values on the *x*-axis represent ADAS13 scores, with higher scores indicating worse performance. Values on the *y*-axis represent ΔFAQ, calculated as the difference between baseline FAQ score and 24-month FAQ score, with higher scores indicating worse functioning at the 24-month visit. Worse ADAS13 scores were associated with greater decline in functioning 24 months after baseline assessment. (**C**) Values on the *x*-axis represent mean SUV of the left angular gyrus, with lower scores indicating lower brain activity. Values on the *y*-axis represent ΔFAQ, calculated as the difference between baseline FAQ score and 24-month FAQ score, with higher scores indicating worse functioning at the 24-month visit. Lower average glucose metabolism in the left angular gyrus was associated with greater decline in functioning 24 months after baseline assessment. EcogSP, Everyday Cognition-Study Partner; FAQ, Functional Activities Questionnaire; SUV, standardized uptake value.

The overall regression model was significant [*F*(12,107) = 16.69, *P *<* *2.2 × 10^−16^] and explained 33.1% of the variance in functional change (Table 4). In this model, only EcogSP-Language, mean left angular gyrus activity, and ADAS13 were significant predictors. Those who scored poorly on EcogSP-Language showed greater decline in functioning at the 24-month follow-up [*F*(1,118) = 71.81, *P *=* *7.8 × 10^−4^; Fig. 3A]. The EcogSP-Language subtest explained 21.1% of the variance in functional change. Those with lower mean activity in the left angular gyrus also showed greater decline at the 24-month follow-up [*F*(1,118) = 58.66, *P *=* *5.8 × 10^−12^; Fig. 3B]. Left angular gyrus activity explained 14.5% of the variance in functional change. Those who scored poorly on the ADAS13 also showed greater decline in functioning at the 24-month follow-up [*F*(1,118) = 1237, *P *<* *2.2 × 10^−16^; Fig. 3C]. The ADAS13 explained 21.3% of the variance in functional change. After accounting for shared variance, these three variables collectively explained 31.7% of the total variance in functional decline [*F*(2,116) = 74.56, *P *<* *2.2 × 10^−16^].

**Table 4:** Results of multiple regression

Variable	Coefficient	SE	95% CI	VIF	*P*-value
ADAS13	0.15	0.03	0.09–0.21	3.13	8.2 × 10^−6*^
Category fluency-animals	−0.04	0.03	−0.10 to 0.01	1.56	0.18
CDR-Sum of boxes	−0.10	0.14	−0.38 to 0.19	1.64	0.50
EcogSP-Divided Attention	−0.46	0.26	−0.98 to 0.05	2.85	0.08
EcogSP-Language	1.55	0.31	0.93–2.17	3.00	2.6 × 10^−6*^
EcogSP-Memory	0.18	0.30	−0.41 to 0.77	3.83	0.55
Mean Left Angular Gyrus	−3.05	0.73	−4.49 to 1.60	1.34	6.28 × 10^−5*^
Left CA2/3 + subiculum	0.00	0.00	−0.001 to 0.001	4.05	0.63
Logical Memory Delayed	0.03	0.03	−0.03 to 0.09	2.09	0.30
MoCA	0.02	0.06	−0.09 to 0.14	2.87	0.68
Right Hippocampal Volume	0.00	0.00	−0.001 to 0.00	4.30	0.55
RAVLT 30 min Delay	0.04	0.03	−0.02 to 0.10	1.98	0.18
Overall Model	0.331^a^		16.69^b^		<2.2 × 10^−16*^

a Model *R*^2^.

b *F*-statistic.

* indicates *P* < 0.05.

ADAS13, Alzheimer’s Disease Assessment Schedule; CDR, Clinical Dementia Rating; EcogSP, Everyday Cognition-Study Partner; MoCA, Montreal Cognitive Assessment; RAVLT, Rey Auditory Verbal Learning Test; SE, standard error; VIF, variance inflation factor

## Discussion

The objective of this study was to use machine learning to reliably predict functional decline within 24 months in cognitively healthy older adults and those with MCI. We first examined clinically relevant modalities (i.e. demographics, MRI, FDG-PET, neurocognitive measures and genetic/fluid-based biomarkers) that predicted functional decline, and then examined individual variables across all modalities. There were two main findings. First, neurocognitive measures demonstrated the best accuracy in predicting functional decline compared to other data modalities (accuracy = 74.2%; AUC = 0.77), followed by FDG-PET (accuracy = 70.8%; AUC = 0.66). Second, when considering the top 15 variables selected by the training sample, only scores on the EcogSP-Language subtest, ADAS13 and mean activity in the left angular gyrus (measured by FDG-PET) explained a significant amount of variance in functional decline in the testing sample.

Results from the SVM algorithms demonstrated that neurocognitive measures predict IADL decline in two years with acceptable discriminability and outperform other measures including neuroimaging, CSF, demographics, and genetics. These results suggest that neurocognitive tests can successfully predict functional decline better than more invasive and costly assessments. Moreover, when examining individual variables’ discriminatory ability across all modalities, 11 of the top 15 predictive variables were neurocognitive. Of these neurocognitive measures, the EcogSP-Language subtest and the ADAS13 demonstrated the strongest association with functional decline in the testing sample. The EcogSP-Language subtest specifically explained 21.1% of the variance. This is consistent with similar work that has identified the Ecog as a predictor of cognitive decline and conversion to MCI or Alzheimer’s disease.^36^ The Ecog is an informant-report questionnaire designed to assess subtle changes in real-world functioning in an older population; it can also be administered as a self-report measure. It has shown high test-retest reliability (*r *=* *0.82), convergent validity with widely used neuropsychological and informant assessments, and external validity in discriminating normal, MCI, and dementia groups.^37^ The EcogSP-Language subtest includes nine items that assess language and semantic memory.^37^ However, it is likely that the EcogSP-Language subtest assesses more complex cognitive abilities than simple language. For example, understanding and communicating in a conversation requires understanding grammatical complexities of language (semantic memory), following and remembering the sequence of remarks made by various speakers (working memory and attention), and retrieving specific event memories to convey thoughts (episodic memory), which decline with age.^38–40^ Traditional language fluency and verbal knowledge tests such as the Category Fluency Test and the American National Adult Reading Test (ANART) were not selected as significant predictors of functional decline in this study, suggesting that EcogSP-Language subtest may be probing more complex information synthesis and processing abilities that underlie functional ability. The Ecog-Language subtest as completed by the study participant also was not selected as a significant predictor. A key feature of this assessment in predicting functional decline may be in the study partner’s assessment of such skills, as study participants may be anosognosic. This is supported by other research that suggests that informant-reported ratings of functional deficits better predict decline and conversion to dementia.^41^

The ADAS13 also demonstrated a significant (*P *<* *2.2 × 10^−16^) association with functional decline and explained 21.3% of the variance in decline. The ADAS13 is a cognitive test (scores between 0 and 85 with higher scores indicating worse cognition) that assesses patients in various domains including learning and memory, language production and comprehension, and constructional and ideational praxis. It is derived from the ADAS11 which was designed to help diagnose mild and moderate Alzheimer’s disease,^42^ and incorporates two additional items (cancellation and delayed free recall) that have been shown to increase the sensitivity of the assessment in differentiating between CN, MCI, and AD.^43^^,^^44^ While this newer version of the ADAS has not yet been well-studied, it has been shown to be predictive of conversion from MCI to Alzheimer’s disease,^45^ help determine Aβ positivity^46^ and identify early stages of Alzheimer’s disease.^47^ Because the ADAS13 is made of multiple domains, it is possible that the results here are being driven by one or several specific areas of cognition. One study showed that five domains were particularly sensitive to cognitive change over time—commands, remember instructions, comprehension, word finding and spoken language.^48^ Our results suggest that the ADAS13 can be a good measure to understand functional decline and the relevant domains may be similar to the construct uncovered by our results. Future work may help determine the importance and relevance of this assessment and potential construct.

Following neurocognitive measures, FDG-PET demonstrated the next best discriminability (AUC = 0.66). FDG-PET has long been used to assess cognitive decline and dementia by serving as a proxy for brain activity. A previous study demonstrated the importance of FDG-PET in predicting functional decline in individuals with MCI.^11^ While Landau and colleagues used a composite measure of the five ROIs also used in this study, the results here specifically identified the left angular gyrus as a significant predictor of functional decline. Further validating the angular gyrus results, the left angular gyrus was a significant predictor in a separate sample that had not been used for variable selection. Regression analysis indicated that activity in the left angular gyrus alone explained 14.5% of the variance in degree of functional change, which underscores the importance of considering the left angular gyrus to develop a more comprehensive understanding of functional decline throughout the ageing process.

The angular gyrus resides in the posterior inferior parietal lobe in Brodmann area 39 and is part of the parietal association cortex.^49^^,^^50^ It serves as a hub linking large scale neural networks, including the default mode network, dorsal attention network, and visual and somatosensory networks, suggesting that this area is critically involved in synthesizing heteromodal information.^51^ In a review integrating a large body of primary research, Seghier et al.^50^ conclude that ‘the [angular gyrus] resembles a cross-modal integrative hub that gives sense and meaning to an event within a contextualized environment, based on prior expectations and knowledge, and towards an intended action’ (p. 52). This observation comes from studies supporting the angular gyrus’s role in semantic associations,^52^ reading and comprehension,^53^ episodic memory retrieval,^54–56^ verbal working memory^57^ and social cognition.^58^ The left angular gyrus may play a more substantial role in *semantic and conceptual knowledge* relative to the right angular gyrus.^49^ Moreover, degeneration specifically in the left angular gyrus has been associated with cognitive decline in neurodegenerative disease.^59^ It is plausible that the ability to carry out IADLs, which depends on the nexus of conceptual knowledge and action sequences, critically relies on left angular gyrus function. The results reported here are consistent with the notion that disruptions in IADLs reflect lower brain activation in this area. While a previous study has demonstrated a cross-sectional link between angular gyrus volume and functional ability,^60^ this study provides evidence for the association between angular gyrus function and future functional decline.

Of the variables identified in the training sample and validated in the testing sample, processing, retrieval, and conceptual integration of semantic information emerged as a construct that may be critical for understanding risk factors of future functional decline. This construct is indicated by the EcogSP-Language and mean SUV in the left angular gyrus. These two measures, one neurocognitive and one neurometabolic, overlap in their purported involvement in integration of conceptual knowledge and action sequences towards a functional goal. The ADAS13 also incorporates semantic language processing items, and future research should examine if these particular items, such as those identified in Dowling et al.,^48^ may be driving this relationship.

Results from the SVM models also show that genetic/fluid-based biomarkers and MRI metrics demonstrate minimal predictive ability, with an AUC of 0.63 and 0.62, respectively. This provides some support for prior research demonstrating their ability to predict functional decline in adults with normal cognition and MCI.^7^^,^^8^ However, when the top 15 variables were validated in an independent sample, no genetic/fluid-based biomarkers or MRI metrics were selected, suggesting that while they may demonstrate predictive ability, this predictive ability may be outranked when other variables are considered. This underscores the importance of considering a large set of variables in a data-driven approach in order to identify those that are most predictive compared to selecting variables *a priori*. While demographic information demonstrated some predictive ability as measured by AUC (0.62), it showed 0% sensitivity, suggesting such variables have minimal utility for studying functional decline over two years.

This study has several limitations. First, it is important to consider potential biases in sample selection. Participants included in ADNI are, on average, highly educated and predominantly white*.* Socioeconomic status has been shown to affect IADL functional status.^1^ Therefore, these results may not generalize to the larger sociocultural context that is representative of individuals within the United States. This study also only examines the predictive ability of various data modalities over 24 months. It cannot be determined whether other data modalities would show greater predictive ability at different timepoints relative to functional decline. Furthermore, these analyses only included data that were already processed through ADNI. Other modalities, such as functional MRI, might improve model performance and predictive power.

It is worth noting that sensitivity in this sample is low. This pattern may be attributed to the multidetermined nature of functional status and decline. Functional ability may be impacted by a number of different factors, including neurodegeneration, physical impairment and medical illness-induced limitations. While we have tried to include a number of diverse variables in the model, it may not be feasible to capture their later effects with baseline variables. For example, an unexpected stroke could severely affect functional decline, but this may not be indicated by any baseline health variable. Additionally, slow physical decline due to illness such as arthritis may impact certain IADL domains such as shopping alone or travelling out of the neighbourhood, but this slow decline may not be evident by baseline status; also, such an illness may not have impacted enough people in the model to affect variable selection. Moreover, research has also demonstrated that the greater the time difference between assessment and prediction, the lower the sensitivity of the model.^18^ Presently, the models developed would require additional information and further validation to improve sensitivity before model deployment in a clinical setting. Nonetheless, the patterns uncovered here shed important light on variables, modalities, and constructs that contribute to our understanding of functional decline and can help guide future research.

## Conclusion

The machine learning approach applied here improves upon previous work examining the ability of neuroimaging, neurocognitive, demographic and genetic/fluid-based biomarkers to predict functional decline two years after baseline assessment. Neurocognitive measures showed the highest accuracy and best discriminative ability, suggesting that a set of inexpensive and non-invasive cognitive assessments can be used to predict independent functioning in lieu of expensive and more invasive measures. Our data-driven approach identified three novel measures (EcogSP-Language, ADAS13 and mean left angular gyrus activity) to predict functional decline, which have not been reported when selecting variables *a priori* or when looking at smaller subsets of variables. Two of these markers (left angular gyrus activity and EcogSP-Language) are purported to be involved in the processing, retrieval, and conceptual integration of semantic information, highlighting this construct in functional ability. The third marker (ADAS13) is a global measure of cognition that may be uniquely designed to target functional decline and certain domains assessed with this tool may be related to the above construct. Future work should consider the utility of the EcogSP-Language, a simple informant report questionnaire, for predicting functional decline in the absence of more invasive procedures such as PET imaging or CSF markers or longer neuropsychological assessments. This questionnaire is non-invasive, time-limited, and cost effective, and the results reported here suggest it may be a useful prediction tool. Future work should further consider the role of the angular gyrus in functional decline in ageing and neurodegenerative disease.

## Supplementary material

Supplementary material is available at *Brain Communications* online.

## Supplementary Material

fcab140_Supplementary_Data

## Abbreviations

Aβ =: amyloid beta
ADAS =: Alzheimer’s Disease Assessment Scale
ADNI =: Alzheimer’s Disease Neuroimaging Initiative
AUC =: area under the curve
EcogSP =: Everyday Cognition-Study Partner
FAQ =: Functional Activities Questionnaire
MCI =: mild cognitive impairment
p-tau =: phosphorylated tau
ROC =: receiver operating characteristic
ROI =: region of interest
SVM =: support vector machine
