# Supplementary material for: Machine learning identifies novel markers predicting functional decline in older adults
Source: Brain Commun. 2021 Jun 26;3(3):fcab140. doi: 10.1093/braincomms/fcab140 (PMC8286801; doi:10.1093/braincomms/fcab140)
Supplement: fcab140_Supplementary_Data [file fcab140_supplementary_data.pdf]

Supplementary Material

Materials and Methods

MRI acquisition and processing

MR images were pre-processed by Mayo Clinic. The University of California San Francisco performed automated cortical reconstruction and volumetric segmentation with FreeSurfer image analysis suite, version 5.1 available for download at (http://surfer.nmr.mgh.harvard.edu/). Scans were processed cross-sectionally using the 2010 Desikan-Killiany atlas. The technical details of these procedures are described in prior publications.[1–14] Briefly, this processing includes motion correction and averaging of multiple volumetric T1 weighted images (when more than one is available)[13], removal of non-brain tissue using a hybrid watershed/surface deformation procedure,[10] automated Talairach transformation, segmentation of the subcortical white matter and deep gray matter volumetric structures (including hippocampus, amygdala, caudate, putamen, ventricles),[6,7] insensitivity normalization,[15] tessellation of the gray matter white matter boundary, automated topology correction,[5,10] and surface deformation following intensity gradients to optimally place the gray/white and gray/cerebrospinal fluid borders at the location where the greatest shift in intensity defines the transition to the other tissue class.[1,2,9]

In order to ensure that high quality data MRI data were collected across sites, all MR images underwent image quality control at the central MRI core laboratory at Mayo Clinic, Rochester Minnesota. For ADNI-GO visits, a phantom scan was collected each day participants were scanned. These scans were checked to ensure proper scanner calibration and for detection of scanner changes indicative of underlying scanner issues. These scans were then used to identify and correct for change in geometric scaling over time, scanner qualification, scanner recalibration, and ongoing scanner quality.[16] For more information, description of the ADNI-GO MRI procedures may be found at http://www.adni-info.org. For ADNI2 visits, the phantom scan was used to certify and update scanners but was no longer collected each day. Improved vendor products were shown to address most of the artifacts that initially warranted a phantom scan. Further, they showed that consistent results could be achieved across different scanners after ADNI 1[17].

Fluorodeoxyglucose-PET (FDG-PET)

Description of FDG-PET acquisition may be found at http://www.adni-info.org. Briefly, patients fasted for at least four hours prior to being injected with ~185MBq of tracer and remained in a dim room for 20-30 minutes following injection. Dynamic 3D scans of six 5-minute frames were collected between 30- and 60-minutes. All PET images underwent quality control checking at the University of Michigan. Images were co-registered to native space, averaged together, aligned, and smoothed to 8mm resolution, which was the lowest resolution of any scanner used to collect data.

Biological samples

To calculate a polygenic hazard score (PHS) for Alzheimer's disease, single nucleotide polymorphisms (SNPs) were identified from a genome-wide association study in the International Genomics of Alzheimer's project (at p < 10-5). Thirty-one SNPs and two *APOE* variants were integrated to create a single hazard score. This score predicts progression to Alzheimer's disease diagnosis as well as age of onset. For a complete description of and methods used to calculate PHS, see Desikan and colleagues.[18]

Analyses of cerebrospinal fluid were performed at the UPenn/ADNI Biomarker Laboratory using Roche Elecsys immunoassay and following Roche Study protocol. The Aβ CSF immunoassay has an upper limit of 1700 pg/mL and lower limit and 200pg/mL; beyond these limits, performance has not been established. Participants with Aβ levels greater than the upper technical limit were truncated to 1700 pg/mL. There were no individuals below the lower technical limit for Aβ or outside of the technical limits for tau (80-1300 pg/mL) or p-tau (8-120 pg/mL).

SMOTE

The Synthetic Minority Oversampling Technique is a method designed to address the imbalance between the number of cases and controls within a dataset. This technique allows the addition of new minority cases into the dataset while preserving variance and without adding redundant information. After identifying a minority case, it then identifies similar minority cases (*k*-nearest neighbors). It generates a new minority case by considering random points between the example and the nearest neighbor to generate additional, plausible, minority cases until the dataset achieves the specified balance.[19] For these analyses, a 50:50 ratio of cases to controls was selected.

Supplemental Table 1

Neurocognitive tests administered

Domain	Test
Complete cognition	Montreal Cognitive Assessment (MoCA)[20]
	Alzheimer's Disease Assessment Scale-Cognitive (ADAS13)[21]
	Clinical Dementia Rating (CDR)[22]
	Everyday Cognition (Ecog)[23]
	Mini-Mental Status Examination (MMSE)[24]
Executive function	Category Fluency Test[25]
	Trail Making Test-Part B[26]
	Clock Drawing Test[27]
Learning and memory	Cognitive Change Index[28]
	Logical Memory Test I and II-delayed paragraph recall[29]
	Rey Auditory Verbal Learning Test (RAVLT)[30]
Language	Boston Naming Test[31]
	American National Adult Reading Test[32]
Processing Speed	Trail Making Test-Part A[26]

Supplementary Table 2

Results using SMOTE

Model	Sensitivity	Specificity	Accuracy	AUC	AUC (95% CI)
Neurocognitive	0.30	0.94	73	0.77	0.68-0.86
Demographics	0.50	1.00	68	0.73	0.63-0.83
MRI	0.15	0.96	69	0.72	0.62-0.83
FDG-PET	0.00	1.00	67	0.70	0.60-0.81
Genetic/fluid-based biomarkers	0.00	1.00	67	0.68	0.57-0.79

Supplementary Figure 1
Correlation plot of selected variables

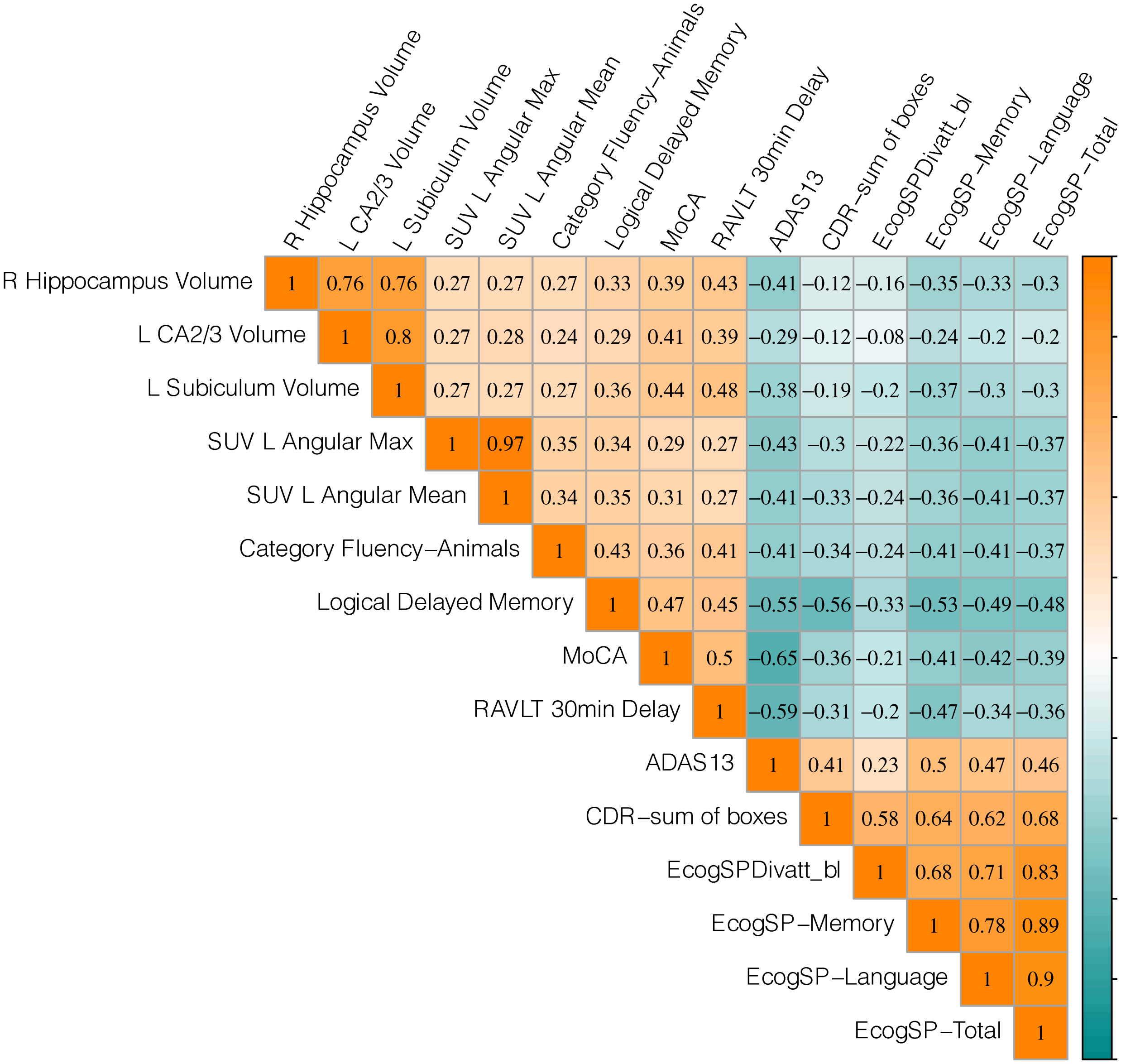

Abbreviations: ADAS13=Alzheimer's Disease Assessment Scale; BL=bilateral; CDR=Clinical Dementia Rating; EcogSP=Everyday cognition–Study Partner; L=left; MoCA=Montreal Cognition Assessment; R=right; RAVLT=Ray Auditory Verbal Learning Test

Supplementary Figure 2
Plot of *c* parameter with repeated k-fold cross-validation

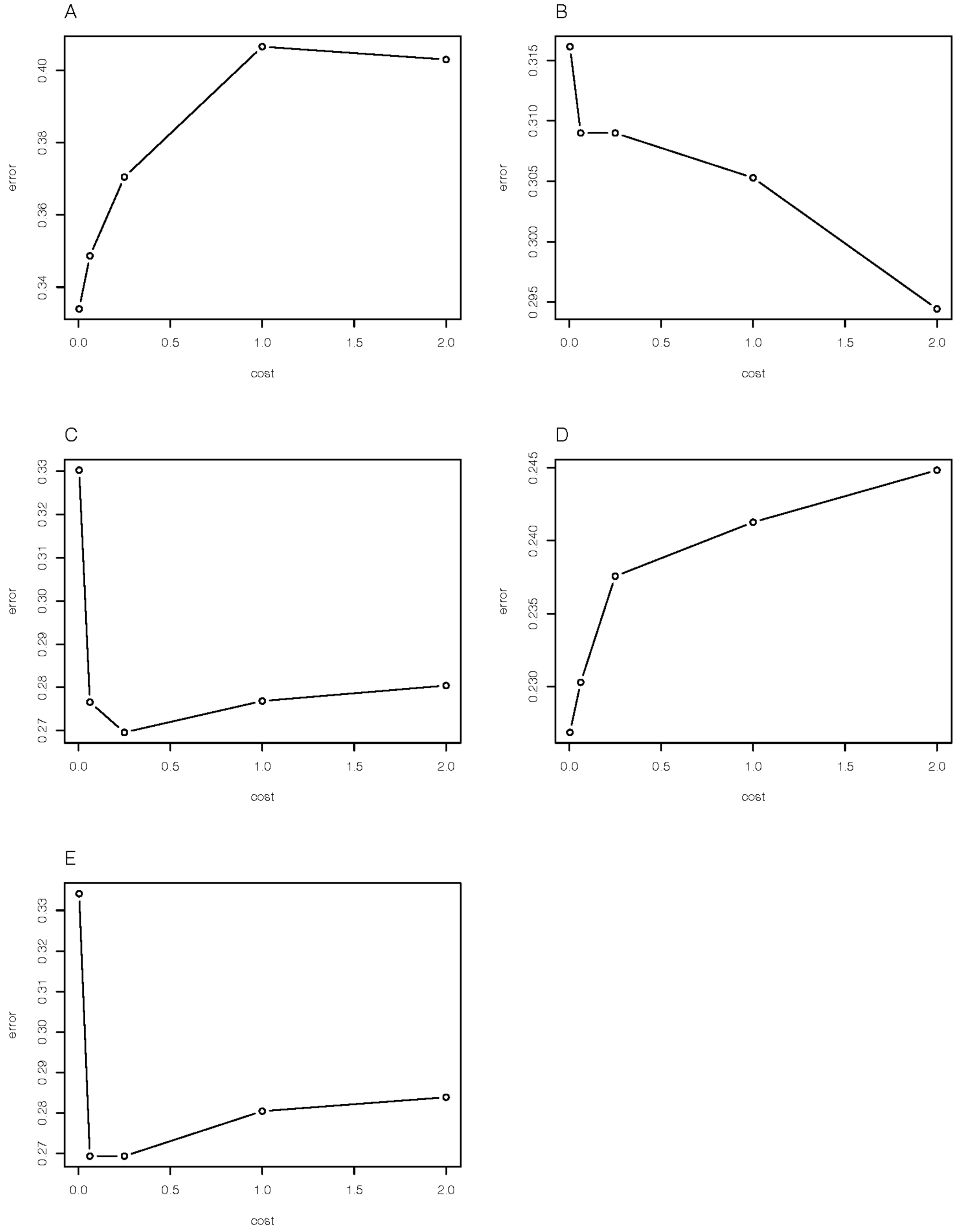

Tuning of *c* parameter occurred in the training sample prior to model evaluation in the testing sample. *C* parameter was tuned with repeated k-fold cross-validation in which k=10 with 50 repeats. Values of c tested were 2^{-8}, 2^{-4}, 2^{-2}, 1, 2, 16.

A Neurocognitive measures
B FDG-PET measures
C Genetics/fluid-based biomarkers
D MRI measures

E Demographic information
Abbreviations: FDG=fluorodeoxyglucose

Supplemental Figure 3
Tripod Checklist

TRIPOD Checklist: Prediction Model Development

Section/Topic	Item	Checklist Item	Page
**Title and abstract**			
Title	1	Identify the study as developing and/or validating a multivariable prediction model, the target population, and the outcome to be predicted.	1
Abstract	2	Provide a summary of objectives, study design, setting, participants, sample size, predictors, outcome, statistical analysis, results, and conclusions.	2-3
**Introduction**			
Background and objectives	3a	Explain the medical context (including whether diagnostic or prognostic) and rationale for developing or validating the multivariable prediction model, including references to existing models.	5-6
	3b	Specify the objectives, including whether the study describes the development or validation of the model or both.	6
**Methods**			
Source of data	4a	Describe the study design or source of data (e.g., randomized trial, cohort, or registry data), separately for the development and validation data sets, if applicable.	6-7
	4b	Specify the key study dates, including start of accrual; end of accrual; and, if applicable, end of follow-up.	6-7
Participants	5a	Specify key elements of the study setting (e.g., primary care, secondary care, general population) including number and location of centres.	6-7
	5b	Describe eligibility criteria for participants.	6-7
	5c	Give details of treatments received, if relevant.	NA
Outcome	6a	Clearly define the outcome that is predicted by the prediction model, including how and when assessed.	7
	6b	Report any actions to blind assessment of the outcome to be predicted.	NA
Predictors	7a	Clearly define all predictors used in developing or validating the multivariable prediction model, including how and when they were measured.	8-9
	7b	Report any actions to blind assessment of predictors for the outcome and other predictors.	NA
Sample size	8	Explain how the study size was arrived at.	6-7
Missing data	9	Describe how missing data were handled (e.g., complete-case analysis, single imputation, multiple imputation) with details of any imputation method.	7
Statistical analysis methods	10a	Describe how predictors were handled in the analyses.	8-9
	10b	Specify type of model, all model-building procedures (including any predictor selection), and method for internal validation.	9-13
	10d	Specify all measures used to assess model performance and, if relevant, to compare multiple models.	12
Risk groups	11	Provide details on how risk groups were created, if done.	NA
**Results**			
Participants	13a	Describe the flow of participants through the study, including the number of participants with and without the outcome and, if applicable, a summary of the follow-up time. A diagram may be helpful.	NA
	13b	Describe the characteristics of the participants (basic demographics, clinical features, available predictors), including the number of participants with missing data for predictors and outcome.	13
Model development	14a	Specify the number of participants and outcome events in each analysis.	Table 1
	14b	If done, report the unadjusted association between each candidate predictor and outcome.	NA
Model specification	15a	Present the full prediction model to allow predictions for individuals (i.e., all regression coefficients, and model intercept or baseline survival at a given time point).	13-15
	15b	Explain how to the use the prediction model.	NA
Model performance	16	Report performance measures (with CIs) for the prediction model.	13; Table 2
**Discussion**			
Limitations	18	Discuss any limitations of the study (such as nonrepresentative sample, few events per predictor, missing data).	20-21
Interpretation	19b	Give an overall interpretation of the results, considering objectives, limitations, and results from similar studies, and other relevant evidence.	20-21
Implications	20	Discuss the potential clinical use of the model and implications for future research.	21
**Other information**			
Supplementary information	21	Provide information about the availability of supplementary resources, such as study protocol, Web calculator, and data sets.	12
Funding	22	Give the source of funding and the role of the funders for the present study.	22-23

We recommend using the TRIPOD Checklist in conjunction with the TRIPOD Explanation and Elaboration document.

1. Dale AM, Sereno MI. Improved localizadon of cortical activity by combining EEG and MEG with MRI cortical surface reconstruction: a linear approach. *J Cogn Neurosci*. 1993;5(2):162-176. doi:10.1162/jocn.1993.5.2.162

2. Dale A, Fischl B, Sereno MI. Cortical surface-based analysis: I. Segmentation and surface reconstruction. *NeuroImage*. 1999;9(2):179-194. doi:10.1006/nimg.1998.0395

3. Fischl B, Sereno MI, Tootell RBH, Dale AM. High-resolution intersubject averaging and a coordinate system for the cortical surface. *Hum Brain Mapp*. 1999;8(4):272-284. doi:10.1002/(SICI)1097-0193(1999)8:4<272::AID-HBM10>3.0.CO;2-4

4. Fischl B, Sereno MI, Dale A. Cortical surface-based analysis: II: inflation, flattening, and a surface-based coordinate system. *NeuroImage*. 1999;9(2):195-207. doi:10.1006/nimg.1998.0396

5. Fischl B, Liu A, Dale AM. Automated manifold surgery: constructing geometrically accurate and topologically correct models of the human cerebral cortex. *IEEE Med Imaging*. 2001;20(1):70-80. doi:10.1109/42.906426

6. Fischl B, Salat DH, Busa E, et al. Whole brain segmentation: automated labeling of neuroanatomical structures in the human brain. *Neuron*. 2002;33:341-355. doi:10.1016/S0896-6273(02)00569-X

7. Fischl B, van der Kouwe A, Destrieux C, et al. Automatically parcellating the human cerebral cortex. *Cereb Cortex*. 2004;14(1):11-22. doi:10.1093/cercor/bhg087

8. Fischl B, Salat DH, van der Kouwe AJW, et al. Sequence-independent segmentation of magnetic resonance images. *NeuroImage*. 2004;23(Supplement 1):S69-S84. doi:10.1016/j.neuroimage.2004.07.016

9. Fischl B, Dale AM. Measuring the thickness of the human cerebral cortex from magnetic resonance images. *Proc Natl Acad Sci U S A*. 2000;97(20):11050-11055. doi:10.1073/pnas.200033797

10. Segonne F, Dale AM, Busa E, et al. A hybrid approach to the skull stripping problem in MRI. *NeuroImage*. 2004;22(3):1060-1075. doi:10.1016/j.neuroimage.2004.03.032

11. Han X, Jovicich J, Salat D, et al. Reliability of MRI-derived measurements of human cerebral cortical thickness: the effects of field strength, scanner upgrade and manufacturer. *NeuroImage*. 2006;32(1):180-194. doi:10.1016/j.neuroimage.2006.02.051

12. Jovicich J, Czanner S, Greve D, et al. Reliability in multi-site structural MRI studies: effects of gradient non-linearity correction on phantom and human data. *NeuroImage*. 2006;30(2):436-443. doi:10.1016/j.neuroimage.2005.09.046

13. Reuter M, Rosas HD, Fischl B. Highly accurate inverse consistent registration: a robust approach. *NeuroImage*. 2010;53(4):1181-1196. doi:10.1016/j.neuroimage.2010.07.020

14. Reuter M, Schmansky NJ, Rosas HD, Fischl B. Within-subject template estimation for unbiased longitudinal image analysis. *NeuroImage*. 2012;61(4):1402-1418. doi:10.1016/j.neuroimage.2012.02.084

15. Sled JG, Zijdenbos AP, Evans AC. A nonparametric method for automatic correction of intensity nonuniformity in MRI data. *IEEE Trans Med Imaging*. 1998;17:87-97. doi:10.1109/42.668698

16. Gunter JL, Bernstein MA, Borowski BJ, et al. Measurement of MRI scanner performance with the ADNI phantom. *Med Phys*. 2009;36(6):2193-2205. doi:10.1118/1.3116776

17. Jack CR, Knopman DS, Jagust WJ, et al. Hypothetical model of dynamic biomarkers of the Alzheimer's pathological cascade. *Lancet Neurol*. 2010;9(1):119-128. doi:10.1016/S1474-4422(09)70299-6

18. Desikan RS, Fan CC, Wang Y, et al. Genetic assessment of age-associated Alzheimer disease risk: Development and validation of a polygenic hazard score. *PLoS Med*. 2017;14(3):e1002258. doi:10.1371/journal.pmed.1002258

19. Chawla NV, Bowyer KW, Hall LO, Kegelmeyer WP. SMOTE: Synthetic Minority Over-sampling Technique. *J Artif Intell Res*. 2002;16:321-357. doi:10.1613/jair.953

20. Nasreddine ZS, Phillips NA, Bédirian V, et al. The Montreal Cognitive Assessment, MoCA: a brief screening tool for mild cognitive impairment. *J Am Geriatr Soc*. 2005;53(4):695-699. doi:10.1111/j.1532-5415.2005.53221.x

21. Rosen WG, Mohs RC, Davis KL. A new rating scale for Alzheimer's disease. *Am J Psychiatry*. 1984;141(11):1356-1364. doi:10.1176/ajp.141.11.1356

22. Berg L. Clinical Dementia Rating (CDR). *Psychopharmacol Bull*. 1988;24(4):637-639.

23. Farias ST, Mungas D, Reed BR, et al. The measurement of everyday cognition (ECog): scale development and psychometric properties. *Neuropsychology*. 2008;22(4):531-544. doi:10.1037/0894-4105.22.4.531

24. Folstein MF, Folstein SE, McHugh PR. "Mini-mental state". A practical method for grading the cognitive state of patients for the clinician. *J Psychiatr Res*. 1975;12(3):189-198. doi:10.1016/0022-3956(75)90026-6

25. Rosen WG. Verbal fluency in aging and dementia. *J Clin Neuropsychol*. 1980;2(2):135-146. doi:10.1080/01688638008403788

26. Reitan RM. Validity of the Trail Making Test as an indicator of organic brain damage. *Percept Mot Skills*. 1958;8(3):271-276.

27. Goodglass H, Kaplan E. *The Assessment of Aphasia and Related Disorders*. Second. Lea & Febiger; 1982.

28. Saykin AJ, Wishart HA, Rabin LA, et al. Older adults with cognitive complaints show brain atrophy similar to that of amnestic MCI. *Neurology*. 2006;67(5):834-842.

29. Wechsler D. Wechsler memory scale-revised. *Psychol Corp*. Published online 1987.

30. Rey A. L'examen psychologique dans les cas d'encéphalopathie traumatique.(Les problems.). *Arch Psychol*. Published online 1941.

31. Kaplan E, Goodglass H, Weintraub S, Goodglass H. *Boston Naming Test*. Lea & Febiger; 1983.

32. Nelson HE, O'Connell A. Dementia: the estimation of premorbid intelligence levels using the New Adult Reading Test. *Cortex J Devoted Study Nerv Syst Behav*. 1978;14(2):234-244. doi:10.1016/s0010-9452(78)80049-5
